# A large intracardiac hydatid cyst with concomitant cervical and hepatic involvement: A case report

**DOI:** 10.1002/ccr3.7307

**Published:** 2023-05-12

**Authors:** Maryam Faramarzpour, Sirous Jafari, Mehrzad Rahmanian, Akram Sardari, Farnoosh Larti

**Affiliations:** ^1^ Department of Cardiology Imam Khomeini Hospital Complex, Tehran University of Medical Sciences Tehran Iran; ^2^ Department of Infectious Disease Imam Khomeini Hospital Complex, Tehran University of Medical Sciences Tehran Iran; ^3^ Department of Cardiac Surgery Imam Khomeini Hospital Complex, Tehran University of Medical Sciences Tehran Iran

**Keywords:** cardiac hydatidosis, cervical mass, interventricular septum, left ventricular hydatid cyst, liver involvement, transthoracic echocardiography

## Abstract

**Key Clinical Message:**

Cardiac hydatidosis is a relatively rare complication of echinococcosis. Understanding the atypical manifestations, potential associated risk factors, and epidemiology leads to optimal and timely management.

**Abstract:**

Cardiac hydatidosis is a relatively rare complication of echinococcosis, with a potentially life‐threatening condition. Here, we reported a large interventricular septal hydatid cyst bulging in the left ventricle accompanied by a huge cervical lamp with recurrent hepatic cysts that underwent cardiac surgery to excise the cyst uneventfully.

## 1 INTRODUCTION

Cystic echinococcosis is a protozoal infectious disease caused by most of the *Echinococcus* species commonly affecting the liver and lungs (two‐thirds and one‐fourth of all reported cases, respectively), with rare involvement of cardiac tissue (less than 2%).^1^

This case highlights the rare concomitant cardiac, cervical, and hepatic involvement with hydatid disease. Together, a good understanding of the atypical manifestations, potential associated risk factors, and epidemiology leads to the optimal and timely management of such patients to minimize worse outcomes.

## 2 CASE PRESENTATION

A 68‐year‐old woman presented to a health care center with a clinical manifestation of a slow‐growing and painless lump on the right side of the cervical region over several weeks. She had no cardiac symptoms. History‐taking revealed working in a sheep farming area in her twenties. Past medical and surgical history included hypertension and hepatic hydatidectomy 2 years ago.

Ultrasound imaging revealed a 47 mm × 75 mm cervical cyst expanded to the superior mediastinum with neither inflammatory response nor spasm of the cervical muscles. The cervical cyst consisted of a bilayer membrane with several membrane‐attached scolices, indicating an active hydatid cyst (cystic echinococcosis type 1, CE1). The lesion was lateral to the common carotid artery and posterior to the internal jugular vein with no cervical lymphadenopathy. Besides, the abdominal ultrasound examination showed multiple active, recurrent hepatic cysts in both the right and left lobes (stage 1), encompassing all liver segments. There was no evidence of biliary dilatation as well.

**FIGURE 1:**
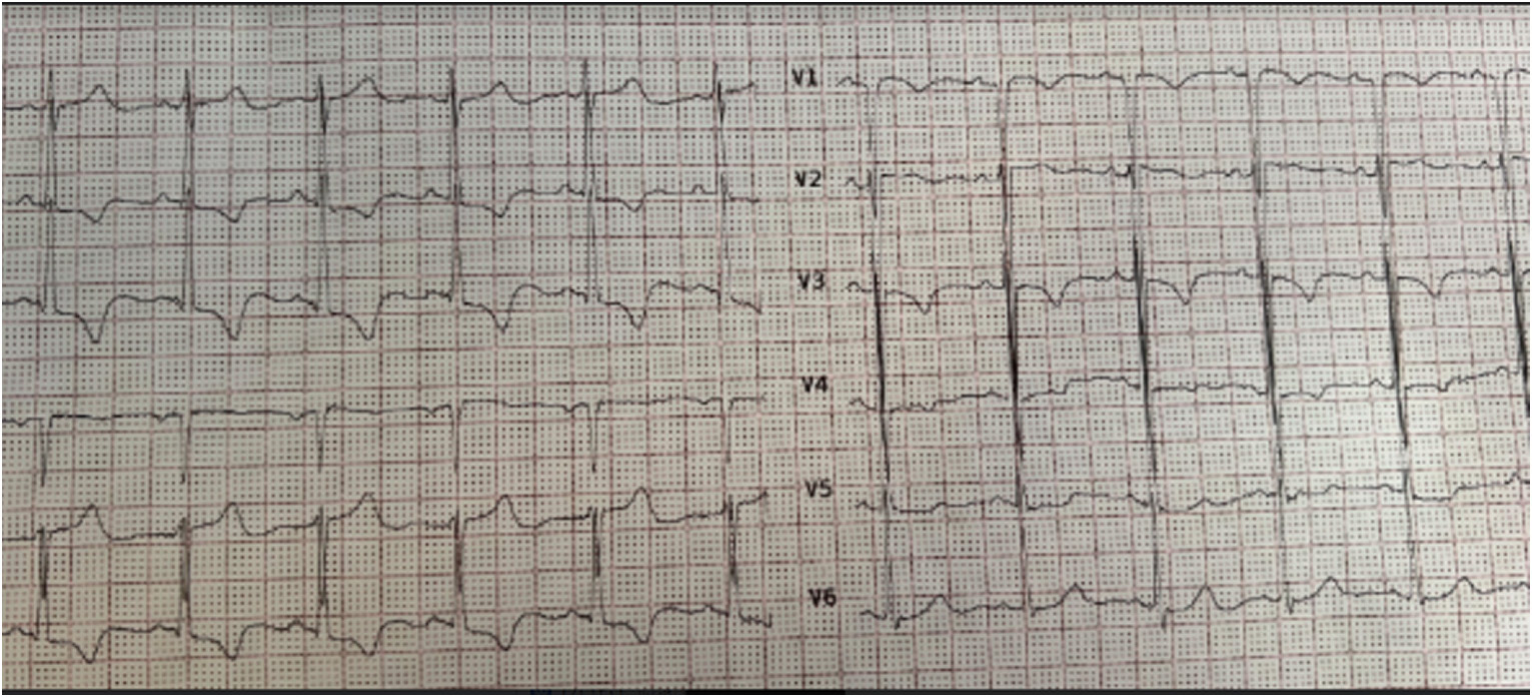
Twelve leads ECG revealed normal sinus rhythm, right axis deviation, with inverted T waves in inferior and V2‐V3 leads.

Electrocardiography revealed normal sinus rhythm, and right axis deviation, with inverted T waves in inferior and V2‐V3 leads. There is no evidence in favor of an atrioventricular block (Figure 1).

**FIGURE 2:**
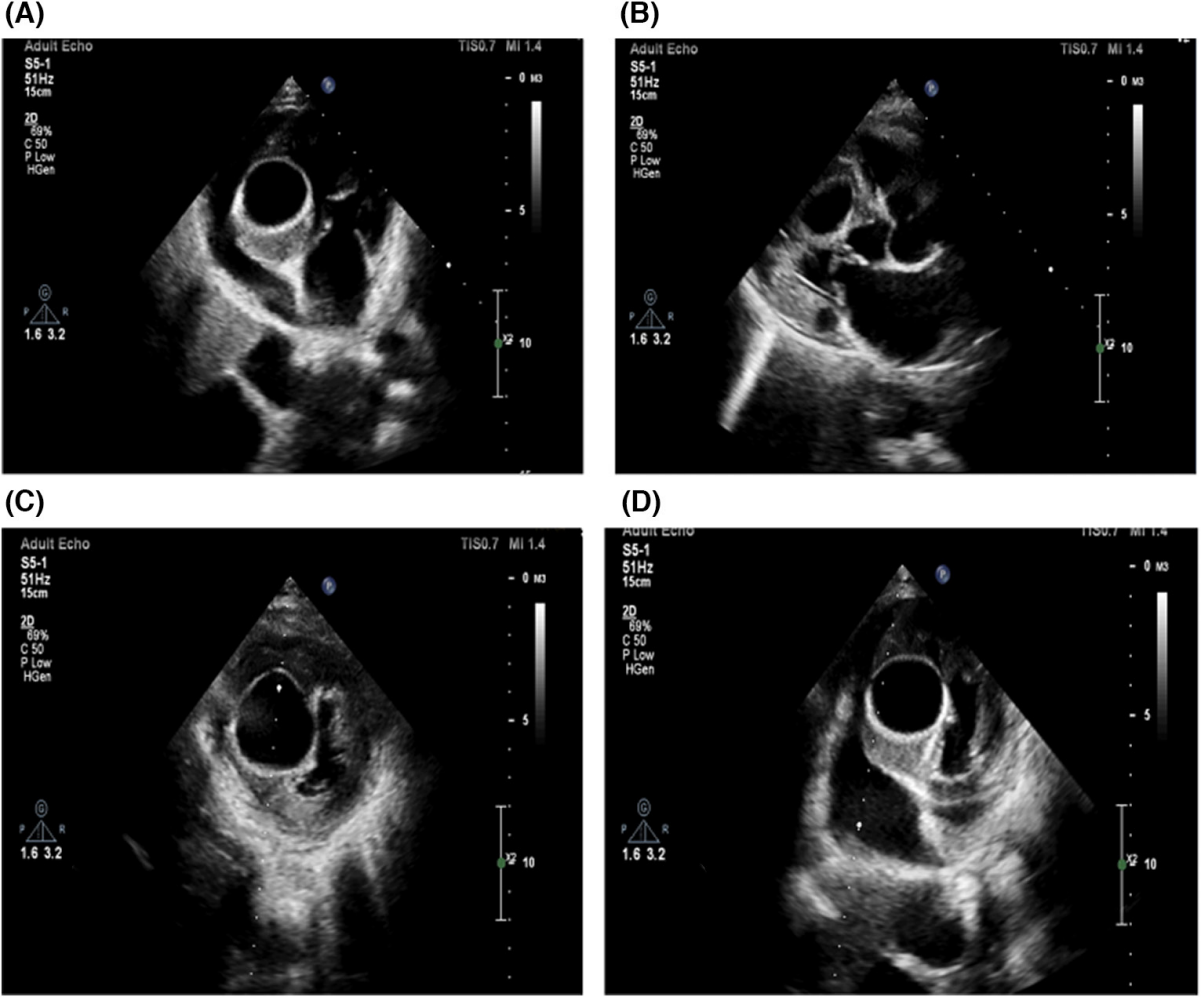
Transthoracic echocardiography shows (A, D) in a four‐chamber view a large, well‐defined intramyocardial cystic lesion (with no obvious septation) in the mid part of the septum with mild compression effect on RVOT without gradient. (B, C) The parasternal long axis and short axis view revealed a cystic lesion.

In the transthoracic echocardiographic (TTE), a bulging and well‐defined echo‐lucent cystic mass in the interventricular septum measuring 33 × 42 mm was detected (Figure 2). A slight compression effect was present on the right ventricle (RV) cavity. The LV size and LV outflow tract (LVOT) were normal, with a mild systolic dysfunction (eyeball estimation of LV ejection fraction = 45%–50%). The valvular functions were normal, with no pericardial effusion. Other echocardiographic findings were unremarkable. The hydatid serology was positive, in which the enzyme‐linked immunosorbent assay (ELISA)‐based qualitative assessment of *E. granulosus* IgG antibodies confirmed the echinococcosis. Treatment with antiprotozoal medication was started. After 2 weeks, the patient underwent coronary angiography, which was normal. Cardiac surgery using cardiopulmonary bypass (CPB) for cystectomy was persuaded to minimize the risk of cyst contents spillage. The CPB technique was established by cannula inserting into the ascending aorta, superior vena cava (SVC), and inferior vena cava (IVC) after the routine median sternotomy.

**FIGURE 3:**
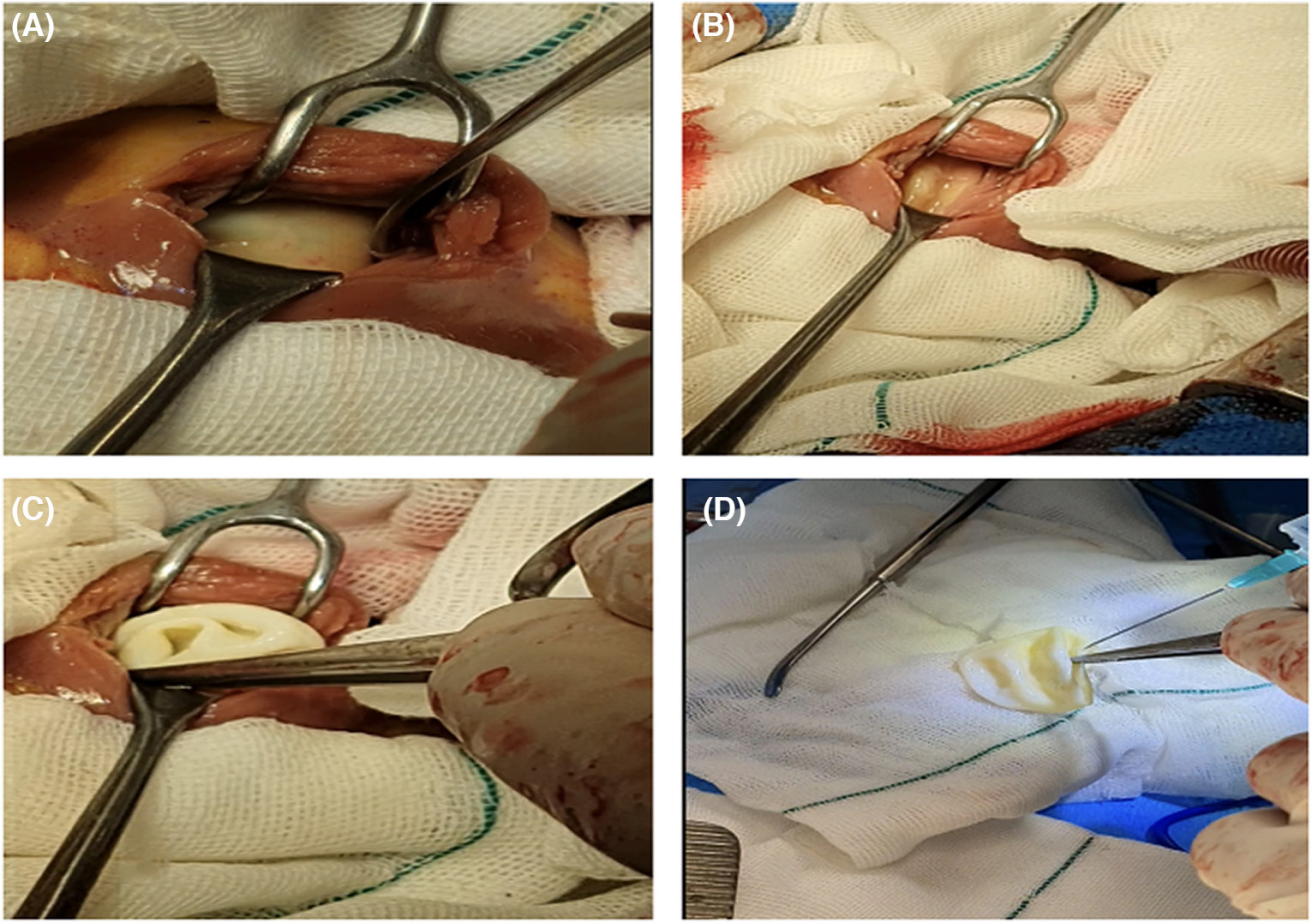
(A) Surgery revealed a complete and clear cardiac cyst (B, C, D). After sterilizing and evacuating the cyst contents, the cardiac cyst was completely resected upon aspiration.

**FIGURE 4:**
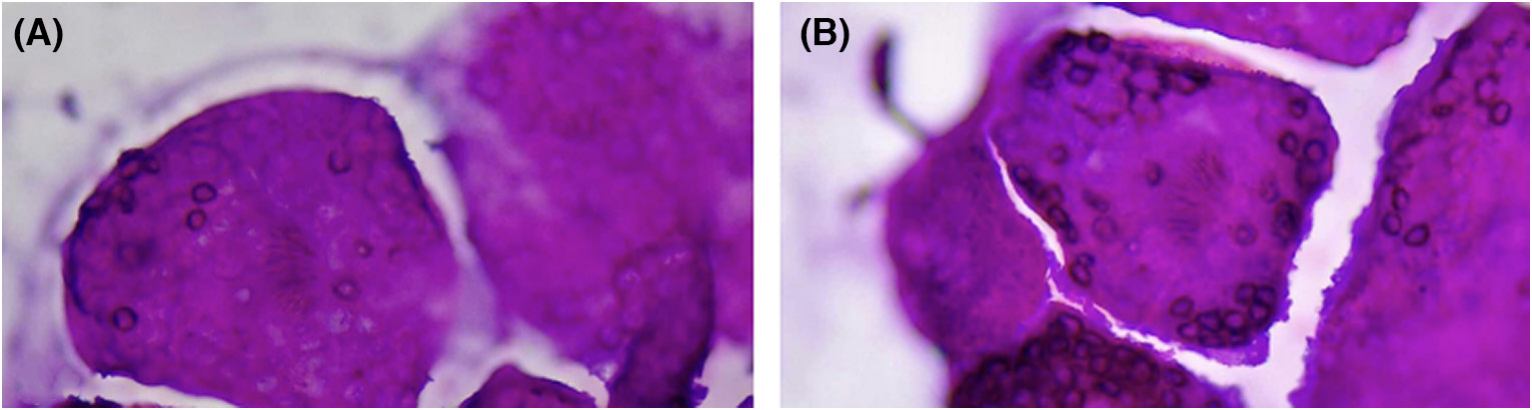
(A, B) Protoscolices of Echinococcus granulosus in cytology, ×400 magnification, Papanicolaou Stain.

Following the cold cardioplegia, the established hypothermia was recorded at 32°C. The outlines of the isolated cardiac cyst seemed to be complete and clear. Conservative procedures were further performed to sterilize and evacuate the cyst contents. The RV cavity was entered, and the cyst was exposed carefully. Thereafter, 10 mL of its contents were aspirated. An equal amount of hypertonic saline (NS 20%) was injected into the cyst, and after several minutes, the exposed cyst was evacuated completely (Figure 3). Following successful excision and secured hemostasis, the cyst specimen, containing 8 mL colorless turbid fluid, was sent to the histopathological examination, which further vouched for the diagnosis of a hydatid cyst (Figure 4). The surgery was successfully terminated with a good recovery phase. A few days later, she was discharged on the anthelminthic drug (albendazole). She had no complications on the first outpatient visit 1 week after discharge. The 3‐month follow‐up was satisfactory.

## 3 DISCUSSION

According to the WHO Informal Working Group on Echinococcosis (WHO‐IWGE) ultrasound classification, hydatid cyst consists of three stages, including active (CE1, CE2, with a high‐risk of rupture), transitional (CE3), and inactive or calcified cysts (CE4, CE5, with a low‐risk of rupture).^1^ The liver and lungs are the most common organs of hydatid involvement. In half of such cardiac cases, multiple organ involvement is also reported.^2^ Since cardiac hydatidosis has the propensity to be enlarged, further pressure over the myocardium results in ventricular dysfunction, mechanical obstruction of atrioventricular valves, and displacement of coronary vessels.^3^ Irrespective of the size and stage, it can cause potentially lethal arrhythmia (ventricular tachycardia),^4^ and high‐grade atrioventricular block.

Peripheral embolization, anaphylactic reactions, and sudden death^5^, ^6^, ^7^ occur following the cyst rupture or left ventricular outflow tract obstruction (LVOTO).^8^ Although most infected patients are asymptomatic, some clinical manifestations, such as dyspnea, chest pain, syncope, and palpitation, can be observed in a minority of cases. In light of the mostly asymptomatic entity of the cardiac hydatid cyst, echocardiography with high sensitivity and specificity is considered an imaging method of choice regarding cyst location, size, scolices presence, and evaluation of the possible pressure on other parts of the heart.^9^ Even so, ultrasound, computed tomography (CT), plain chest X‐ray, and cardiac magnetic resonance (CMR) are known as other valuable diagnostic measures.^10^, ^11^ Beyond the imaging tools, serology and pathohistological examinations would be helpful aids in confirming disease diagnosis. Hitherto, complete surgical excision remains a preferred therapeutic option. Moreover, albendazole/mebendazole^12^ and praziquantel are prescribed as adjuvant pharmacotherapy.^1^

Although most cardiac hydatic cysts in the literature are reported in young patients, we reported an old lady (68 y/o) with a recurrent hepatic hydatidosis accompanied by a huge homogenous cystic mass in the cervical and intracardiac regions. In our case, the cystic lesions near the portal venous confluence and the left portal vein may be the leading cause of extrahepatic hydatidosis. It is worth noting that the multiplicity and dispersion of the lesions, typical imaging findings, a history of husbandry procedures, a history of hepatic cysts, geographic location, and positive result of serologic test strongly established the hydatid cyst diagnosis. A previous study represented a 70 y/o female with no history of being in a sheep rising area with signs in favor of right heart failure and cardiac hydatidosis complicated hydatid cyst and pretamponade.^13^ Shojaei et al., in Iran, also reported a cardiac HC in a 70‐year‐old farmer with dyspnea. The isolated lesion was diagnosed by echocardiography and further confirmed with cardiac MRI. Despite successful surgical excision, he died due to a progressive arrhythmia.^14^ Another report in Iran has also documented an echinococcal infection involving an intramyocardial multicystic lump in the posterolateral and basal inferoseptal segments of LV in a 57‐year‐old farmer man referred with chest pain, and diagnosed by echocardiography, CMR, and positive ELISA‐based serologic test. In contrast to our finding, EKG examination showed pathological Q and negative T waves. Similarly, surgery was the treatment of choice, followed by albendazole as a complementary therapy.^3^

In conclusion, a good understanding of the atypical manifestations, potential associated risk factors, and epidemiology leads to the optimal and timely management of patients with rare echinococcosis to minimize worse outcomes.

## AUTHOR CONTRIBUTIONS

**Maryam Faramarzpour:** Project administration; resources; writing – original draft. **Sirous Jafari:** Project administration; resources. **Mehrzad Rahmanian:** Resources. **Akram Sardari:** Writing – original draft. **Farnoosh Larti:** Conceptualization; resources; writing – review and editing.

## FUNDING INFORMATION

No funding was received for this study.

## CONFLICT OF INTEREST STATEMENT

No conflict of interest was present to be disclosed.

## CONSENT

Written informed consent was obtained from the patient regarding this research process.

## Data Availability

The data of this study are available for further assessment.

## References

[1] Ameen A , Hilal K , Shaikh A , Khan F , Fatimi S . Cardiac hydatid cyst presenting as ventricular arrhythmia: a case report. Egypt Heart J. 2021;73(1):105. 34874501 10.1186/s43044-021-00231-z PMC8651847

[2] Separovic Hanzevacki J , Gasparovic H , Reskovic Luksic V , Ostojic Z , Biocina B . Staged management of a giant cardiac hydatid cyst: a case report. BMC Infect Dis. 2018;18(1):694. 30587137 10.1186/s12879-018-3599-2 PMC6307286

[3] Firouzi A , Neshati Pir Borj M , Alizadeh GA . Cardiac hydatid cyst: a rare presentation of echinococcal infection. J Cardiovasc Thorac Res. 2019;11(1):75‐77. 31024677 10.15171/jcvtr.2019.13 PMC6477106

[4] Dong Z , Yusup M , Lu Y , Tang B . Hydatid cyst of the heart as a rare cause of arrhythmia: a case report and review of published reports. Hear Case Rep. 2022;8:458‐462. 10.1016/j.hrcr.2022.04.004 PMC9237349 35774212

[5] Mesrati MA , Mahjoub Y , Abdejlil NB , et al. Case report: sudden death related to unrecognized cardiac hydatid cyst. F1000Research. 2020;9:286. 33500772 10.12688/f1000research.23277.1 PMC7814283

[6] Bajdechi M , Manolache D , Tudor A , Orghidan M , Gurghean A . Cardiac hydatid cysts in a young man: a case report and a literature review. Exp Ther Med. 2022;24(3):1‐10. 10.3892/etm.2022.11487 PMC9366287 35978922

[7] Manuel V , Neto MP , Garcia ZS , Delgado C . Swiss cheese heart: cardiac hydatid cysts. Can J Cardiol. 2022;39:87‐88. 36336306 10.1016/j.cjca.2022.10.031

[8] Oner T , Korun O , Celebi A . A cardiac hydatid cyst mimicking a pericardial tumour in a paediatric case. Cardiol Young. 2019;29(2):244‐246. 30511599 10.1017/S1047951118002032

[9] Gupta A , Mishra SC , Jaiswal S , Pande S . Intracardiac hydatid cyst located in right ventricular outflow tract: a rare site. Indian J Thorac Cardiovasc Surg. 2021;37(5):588‐590. 34511771 10.1007/s12055-021-01165-6 PMC8387540

[10] Al‐Dairy A , Abo KR . Surgical excision of a cardiac hydatid cyst from the right ventricle in a child. Clin Case Rep. 2021;9(8):e04714. 34466264 10.1002/ccr3.4714 PMC8385462

[11] Kankilic N , Aydin MS , Günendi T , Göz M . Unusual hydatid cysts: cardiac and pelvic‐Ilio femoral hydatid cyst case reports and literature review. Braz J Cardiovasc Surg. 2020;35:465‐472. 32865381 10.21470/1678-9741-2019-0153 PMC7454634

[12] AlShamlan RA , Almousa AM , Al Saeed MJ , Al‐Dera FH , Alobaydun MA . Cardiac hydatid cyst successfully managed with albendazole: a case report. Cureus. 2019;11(12):e6405. 31970035 10.7759/cureus.6405 PMC6964963

[13] El Boussaadani B , Regragui H , Bouhdadi H , et al. Primary cardiac hydatid cyst presenting with massive pericardial effusion: a case report. Egypt Heart J. 2020;72(1):1‐4. 10.1186/s43044-020-00085-x PMC7431496 32804331

[14] Shojaei E , Yassin Z , Rezahosseini O . Cardiac hydatid cyst: a case report. Iran J Public Health. 2016;45(11):1507‐1510. 28028503 PMC5182260

